# A dynamic online nomogram for predicting death in hospital after aneurysmal subarachnoid hemorrhage

**DOI:** 10.1186/s40001-023-01417-8

**Published:** 2023-10-12

**Authors:** Tian Li, Dongzhou Zhuang, Yong Xiao, Xiaoxuan Chen, Yuan Zhong, Xurong Ou, Hui Peng, Shousen Wang, Weiqiang Chen, Jiangtao Sheng

**Affiliations:** 1 Department of Microbiology and Immunology, Guangdong Provincial Key Laboratory of Infectious Disease and Molecular Immunopathology, Shantou University Medical College, 22 Xinling Road, Shantou, 515000 Guangdong China; 2 Department of Neurosurgery, Fuzong Clinical Medical College of Fujian Medical University, 900 Hospital, Fuzhou, 350025 China; 3 Department of Neurosurgery, First Affiliated Hospital, Shantou University Medical College, 57 Changping Road, Shantou, 515000 Guangdong China; 4 Department of Neurosurgery, Affiliated Jieyang People’s Hospital of Sun Yat-sen University, 107 Tianfu Road, Jieyang, 522000 China

**Keywords:** Multiplication of neutrophil and monocyte counts, Aneurysmal subarachnoid hemorrhage, Nomogram, Inflammation, Outcome

## Abstract

**Background:**

This study aimed to validate the efficacy the multiplication of neutrophils and monocytes (MNM) and a novel dynamic nomogram for predicting in-hospital death in patients with aneurysmal subarachnoid hemorrhage (aSAH).

**Methods:**

Retrospective study was done on 986 patients with endovascular coiling for aSAH. Independent risk factors associated with in-hospital death were identified using both univariate and multivariate logistic regression analysis. In the development cohort, a dynamic nomogram of in-hospital deaths was introduced and made available online as a straightforward calculator. To predict the in-hospital death from the external validation cohort by nomogram, calibration analysis, decision curve analysis, and receiver operating characteristic analysis were carried out.

**Results:**

72/687 patients (10.5%) in the development cohort and 31/299 patients (10.4%) in the validation cohort died. MNM was linked to in-hospital death in univariate and multivariate regression studies. In the development cohort, a unique nomogram demonstrated a high prediction ability for in-hospital death. According to the calibration curves, the nomogram has a reliable degree of consistency and calibration. With threshold probabilities between 10% and 90%, the nomogram’s net benefit was superior to the basic model. The MNM and nomogram also exhibited good predictive values for in-hospital death in the validation cohort.

**Conclusions:**

MNM is a novel predictor of in-hospital mortality in patients with aSAH. For aSAH patients, a dynamic nomogram is a useful technique for predicting in-hospital death.

## Introduction

Aneurysmal subarachnoid hemorrhage (aSAH) is a worldwide health concern that seriously threatens the life of patients [1]. Although aneurysms are effectively controlled by surgery for rebleeding, let there is a mortality rate of about 33% [2]. Recent studies have demonstrated a correlation between inflammation and unfavorable prognostic outcomes in cerebrovascular diseases, specifically stroke [3–5]. According to current reports, leukocyte count [6–9], monocyte count [10, 11], neutrophil-to-lymphocyte ratio (NLR) [1, 12, 13], lymphocyte-to-monocyte ratio [14, 15], and C-reactive protein [16, 17] are inflammatory indicators after aSAH. As a result, there is a strong correlation between hemorrhagic inflammation and neurological complications [18].

Inflammation of the central nervous system after aSAH leads to early brain damage and contributes significantly to delayed cerebral ischemia and cerebral vasospasm (CVS) [19]. Several studies in humans and animals have suggested that neutrophils and monocytes mediate the early inflammatory response and influence the prognosis of patients with aSAH [10, 20]. Several studies have shown that MNM detects cervical cancer early and predicts epithelial ovarian cancer development [21, 22]. Inconceivably, MNM has only been discussed in a few studies in other fields as another important indicator of inflammation. Despite this, it is unclear what role MNM plays in patients with aSAH in terms of prognosis. Therefore, this study aimed to investigate the relationship between MNM and in-hospital death following aSAH and to develop a new nomogram to predict in-hospital death after aSAH.

## Patients and methods

### Patient population

Patients of aSAH who underwent endovascular coil therapy from January 1, 2014 to September 30, 2019 at the First Affiliated Hospital of Shantou University Medical College were included in the retrospective cohort. The external validation cohort included patients from Jieyang People’s Hospital affiliated of Sun Yat-sen University between December 18, 2019 and May 1, 2021. The study was approved by Jieyang People’s Hospital affiliated of Sun Yat-sen University (No. 2021097) and the First Affiliated Hospital of Shantou University Medical College (No. B-2021-244). The study was conducted within the framework of the Declaration of Helsinki (revised by Brazil in 2013). All methods were performed within the framework of the guidelines and regulations. All patients gave informed consent to participate in the study.

The inclusion criteria were as follows: (1) spontaneous aSAH observed by head CT or lumbar puncture at admission; (2) confirmation of the presence of an intracranial aneurysm by CT angiography (CTA), magnetic resonance angiography (MRA) or digital subtraction angiography (DSA); (3) blood samples from all patients upon admission; and (4) patients undergoing endovascular embolization in our hospital. Patients caused by the following were excluded: (1) other reasons (hypertensive cerebral hemorrhage, vascular malformation, tumor, stroke); (2) a history of aSAH or other serious cardiovascular and cerebrovascular diseases; or (3) incomplete clinical data to prevent any interference.

### Preoperative examination and treatment

Routine preoperative examinations include head computed tomography, coagulation function, emergency biochemistry, and blood routine examination. The preoperative diagnosis was according to the results of the DSA or CTA. According to the patient's condition, lumbar puncture, ventral drainage, cerebrospinal fluid drainage, or decompressive craniectomy were chosen after intravascular treatment. All patients with aSAH were treated according to stroke-related guidelines.

### Clinical characteristics

In this study, a total of 986 patients who met the inclusion criteria were included in the study. 687 patients from the First Affiliated Hospital of Shantou University Medical College and 299 patients from Jieyang People’s Hospital of Sun Yat-sen University were categorized into the development cohort and validation cohort, respectively. Baseline clinical and demographic variables were collected after admission, including age, gender, admission blood pressure, smoking and alcohol use history, hypertension, and diabetes mellitus, modified Fisher grade (mFS grade), Hunt–Hess grade, Level on admission Glasgow Coma Scale (GCS) score, National Institutes of Health Stroke Scale (NIHSS) score, serum leukocyte count (reference range, 3.5–9.5 × 10^9^ cells/L), neutrophils count (reference range, 1.8–6.4 × 10^9^ cells/L), monocytes count (reference range, 0.1–0.6 × 10^9^ cells/L), lymphocytes count (reference range, 1.1–3.2 × 10^9^ cells/L), platelets count (reference range, 100–300 × 10^9^/L), serum calcium level and blood glucose level. MNM was calculated as the multiplication of neutrophil and monocyte counts. Finally, we faithfully recorded the patient’s living status and the exact time of death.

### Statistical analysis

R (version 4.1.0; R Foundation, Vienna, Austria) and its corresponding software packages (R Foundation for Statistical Computing), as well as the Statistical Products and Services Solution (version 26; IBM Corp., Armonk, New York, USA), were used for the statistical analysis [23–26]. Variables with continuous data were represented by medians with quartile spacing (IQR), and categorical variables were represented by counts with percentages. Between-group differences were analyzed to reflect patient differences in demographic, clinical, and radiological characteristics and inflammatory markers. Logistic regression analysis was used to select the potential risk factors of in-hospital death, and multiple regression analysis was performed to analyze the independent risk factors of in-hospital death using the forward selection method.

The relationship between MNM and in-hospital death was examined using two linear regression models and smoothing functions [27]. Based on multiple logistic regression, a prediction model was established to predict in-hospital death: a basic model, including independent risk factors, such as Hunt–Hess grade, mFS grade, NIHSS score, and glucose, and a complete model, consisting of the basic model and MNM. Area under the receiver operating characteristic curve (ROC) and decision curve analysis (DCA) have been used to determine the efficacy and clinical benefit of predictive models [28, 29]. DCA can be performed to compare the net benefits of the models and to gain insight into the range of predicted risks that have higher net benefits than classifying all patients as having outcomes or classifying none as having outcomes [30].

Based on the full model, an in-hospital death prediction nomogram was constructed. A nomogram is a multiple regression analysis based on multiple clinical indicators or biological attributes, using line segments with scores to predict death probability. Using the graph calibration method, the prediction calibration curve can be compared to the standard curve to determine how well the nomogram predicts. The closer the calibration curve is to the standard curve, the more accurate the prediction is [31]. For clinical accuracy, we reassessed the AUC for predicting unfavorable prognosis based on each patient’s total score calculated from the nomogram. Kaplan–Meier curve is a common method for survival analysis, which mainly analyzes the impact of a single factor on survival and is used to estimate the survival rate of patients.

## Results

### Patient characteristics

Among 986 patients who met the inclusion criteria, no significant difference was found in the distribution of variables between the development and validation cohorts (Table 1). The differences between the survival and death groups are shown in Table 2. Level on GCS score, Hunt–Hess grade, mFS grade, NIHSS score, blood glucose, and levels of inflammatory markers such as leukocytes, neutrophils, monocytes, NLR, MLR, and MNM showed significant differences between the survival group and the death group (Table 2).

**Table 1:** Baseline clinical characteristics of 986 patients with Subarachnoid hemorrhage Between the development group and the validation group

Variable	Development (*n* = 687)	Validation (*n* = 299)
Gender	265 (38.57%)	117 (39.13%)
Age, years	58 (50–65)	60 (51–66)
Level on GCS score		
Mild (13–15 points)	455 (66.23%)	143 (47.83%)
Moderate (9–12 points)	94 (13.68%)	72 (24.08%)
Severe (3–8 points)	138 (20.09%)	84 (28.09%)
Hunt–Hess grade		
I	137 (19.94%)	42 (14.05%)
II	131 (19.07%)	85 (28.43%)
III	266 (38.72%)	90 (30.10%)
IV	130 (18.92%)	63 (21.07%)
V	23 (3.35%)	19 (6.35%)
mFS grade		
0	3 (0.44%)	4 (1.34%)
I	107 (15.57%)	24 (8.03%)
II	347 (50.51%)	146 (48.83%)
III	138 (20.09%)	81 (27.09%)
IV	92 (13.39%)	44 (14.72%)
NIHSS score	2 (0–13)	4 (1–13)
Hypertension	270 (39.30%)	150 (50.17%)
MAP, mmHg	136 (120–153)	111 (101–124)
Diabetes	49(7.13%)	19 (6.35%)
Smoking	44 (6.40%)	22 (7.36%)
Drink abuse	34 (4.95%)	22 (7.36%)
Blood glucose	8.40 (7.00–10.79)	8.74 (7.30–10.57)
Serum calcium level	2.27 (2.18–2.36)	2.17 (2.08–2.24)
Leukocyte count	13.84 (10.67–17.01)	13.50 (10.75–16.63)
Neutrophil count	11.61 (8.25–14.64)	11.08 (7.96–14.01)
Lymphocyte count	1.36 (0.91–2.00)	1.58 (0.98–2.28)
Monocyte count	0.56 (0.38–0.78)	0.64 (0.42–0.89)
Platelet count	236 (197–275)	239 (201–289)
NLR	8.81 (4.98–14.03)	7.49 (3.77–12.06)
MLR	0.38 (0.26–0.58)	0.40 (0.25–0.64)
PLR	169.52 (115.19–254.10)	152.35 (100.45–229.63)
NWR	0.85 (0.78–0.90)	0.82 (0.73–0.88)
MNM	5.70 (3.25–10.21)	6.72 (3.71–11.15)
mRS (3–6 points)	264 (38.43%)	117 (39.13%)
Death	72 (10.48%)	31 (10.37%)

*GCS* Glasgow Coma Scale, *MAP* mean arterial pressure, *mFS grade* modified Fisher grade, *NIHSS* National Institutes of Health Stroke Scale, *NLR* neutrophil-to-lymphocyte ratio, *MLR* monocyte-to-lymphocyte ratio, *PLR* platelet-to-lymphocyte ratio, *NWR* neutrophil-to-leukocyte ratio, *MNM* multiplication to neutrophil and monocyte counts, *mRS* modified Rankin Scale

**Table 2:** Differences between with Survival group and the Death group (univariate analysis)

Variable	Development cohort (*n* = 687)			Validation cohort (*n* = 299)		
	Survival group (*n* = 615)	Death group (*n* = 72)	*p* value	Survival group (*n* = 268)	Death group (*n* = 31)	*p *value
Sex	241 (39.19%)	24 (33.33%)	0.402	100 (37.31%)	17 (54.84%)	0.089
Age	58 (50–65)	59.5 (53.5–65)	0.202	60 (51–66)	64 (55–66)	0.127
Level on GCS score			< 0.001			< 0.001
Mild (13–15 score)	428 (69.59%)	27 (37.50%)		138 (51.49%)	5 (16.13%)	
Moderate (9–12 score)	83 (13.50%)	11 (15.28%)		67 (25.00%)	5 (16.13%)	
Severe (3–8 score)	104 (16.91%)	34 (47.22%)		63 (23.51%)	21 (67.74%)	
MAP, mmHg	135.33 (120.00–151.33)	144.33 (124.83–156.67)	0.035	110.67 (101.25–123.33)	113.33 (102.33–130.33)	0.429
Hypertension	235 (38.21%)	35 (48.61%)	0.058	136 (50.78%)	14 (45.16%)	0.690
Diabetes	41 (6.67%)	8 (11.11%)	0.252	15 (5.60%)	4 (12.90%)	0.120
Hunt–Hess grade			< 0.001			< 0.001
I	128 (20.81%)	9 (12.50%)		41 (15.30%)	1 (3.23%)	
II	124 (20.16%)	7 (9.72%)		82 (30.60%)	3 (9.68%)	
III	247 (40.16%)	19 (26.39%)		85 (31.72%)	5 (16.13%)	
IV	102 (16.59%)	28 (38.89%)		49 (18.28%)	14 (45.16%)	
V	14 (2.28%)	9 (12.50%)		11 (4.10%)	8 (25.81%)	
mFS grade			< 0.001			< 0.029
0	3 (0.49%)	0 (0.00%)		3 (1.12%)	1 (3.23%)	
I	101 (16.42%)	6 (8.33%)		24 (8.96%)	0 (0%)	
II	323 (52.52%)	24 (33.33%)		40 (14.93%)	4 (12.90%)	
III	110 (17.89%)	28 (38.89%)		66 (24.63%)	15 (48.39%)	
IV	78 (12.68%)	14 (19.44%)		135 (50.37%)	11 (35.48%)	
NIHSS score	2 (0–9)	24.5 (2–35)	< 0.001	3 (1, 8)	26 (7, 35.5)	< 0.001
Smoking	43 (7.15%)	1 (1.39%)	0.074	21 (7.84%)	1 (3.23%)	0.197
Drink abuse	33 (5.37%)	1 (1.39%)	0.244	21 (7.84%)	1 (3.23%)	0.197
Blood glucose	8.20 (6.90–10.34)	10.70 (8.31–13.17)	< 0.001	8.52 (7.12–10.29)	10.85 (8.67–16.24)	< 0.001
Serum calcium level	2.27 (2.18–2.35)	2.30 (2.19–2.39)	0.205	2.17 (2.08–2.24)	2.17 (2.08–2.24)	0.880
Leukocyte count	13.34 (10.31–16.50)	16.93 (14.46–20.91)	< 0.001	13.09 (10.23–16.05)	17.94 (15.79–21.14)	< 0.001
Neutrophil count	11.12 (8.04–14.24)	14.31 (12.31–18.64)	< 0.001	10.82 (7.89–13.61)	14.08 (11.78–18.57)	< 0.001
Lymphocyte count	1.35 (0.92–1.98)	1.42 (0.86–2.14)	0.949	1.58 (0.94–2.26)	1.71 (1.37–2.60)	0.102
Monocyte count	0.54 (0.36–0.76)	0.72 (0.52–0.98)	< 0.001	0.59 (0.41–0.80)	1.39 (0.90–1.63)	< 0.001
Platelet count	235 (197–275)	243 (196.5–271)	0.498	239.50 (199–291.50)	239 (211–257.50)	0.577
NLR	8.53 (4.84–13.63)	12.63 (6.15–17.20)	0.003	7.24 (3.76–12.04)	9.02 (4.14–12.00)	0.636
MLR	0.38 (0.25–0.55)	0.56 (0.32–0.81)	< 0.001	0.38 (0.24–0.60)	0.88 (0.33–1.30)	< 0.001
PLR	168.21 (118.11–251.57)	172.37 (103.88–265.11)	0.852	153.36 (105.22–241.01)	139.34 (89.65–179.16)	0.060
NWR	0.85 (0.77–0.9)	0.87 (0.81–0.9)	0.044	0.82 (0.73–0.89)	0.80 (0.73–0.86)	0.207
MNM	5.35 (3.11–9.35)	10.58 (6.58–16.66)	< 0.001	6.19 (3.50–10.17)	21.44 (12.71–25.97)	< 0.001

*GCS* Glasgow Coma Scale, *MAP* mean arterial pressure, *mFS grade* modified Fisher grade, *NIHSS* National Institutes of Health Stroke Scale, *NLR* neutrophil-to-lymphocyte ratio, *MLR* monocyte-to-lymphocyte ratio, *PLR* platelet-to-lymphocyte ratio, *NWR* neutrophil-to-leukocyte ratio, *MNM* multiplication to neutrophil and monocyte counts

### MNM predicts in-hospital death

**Fig. 1:**
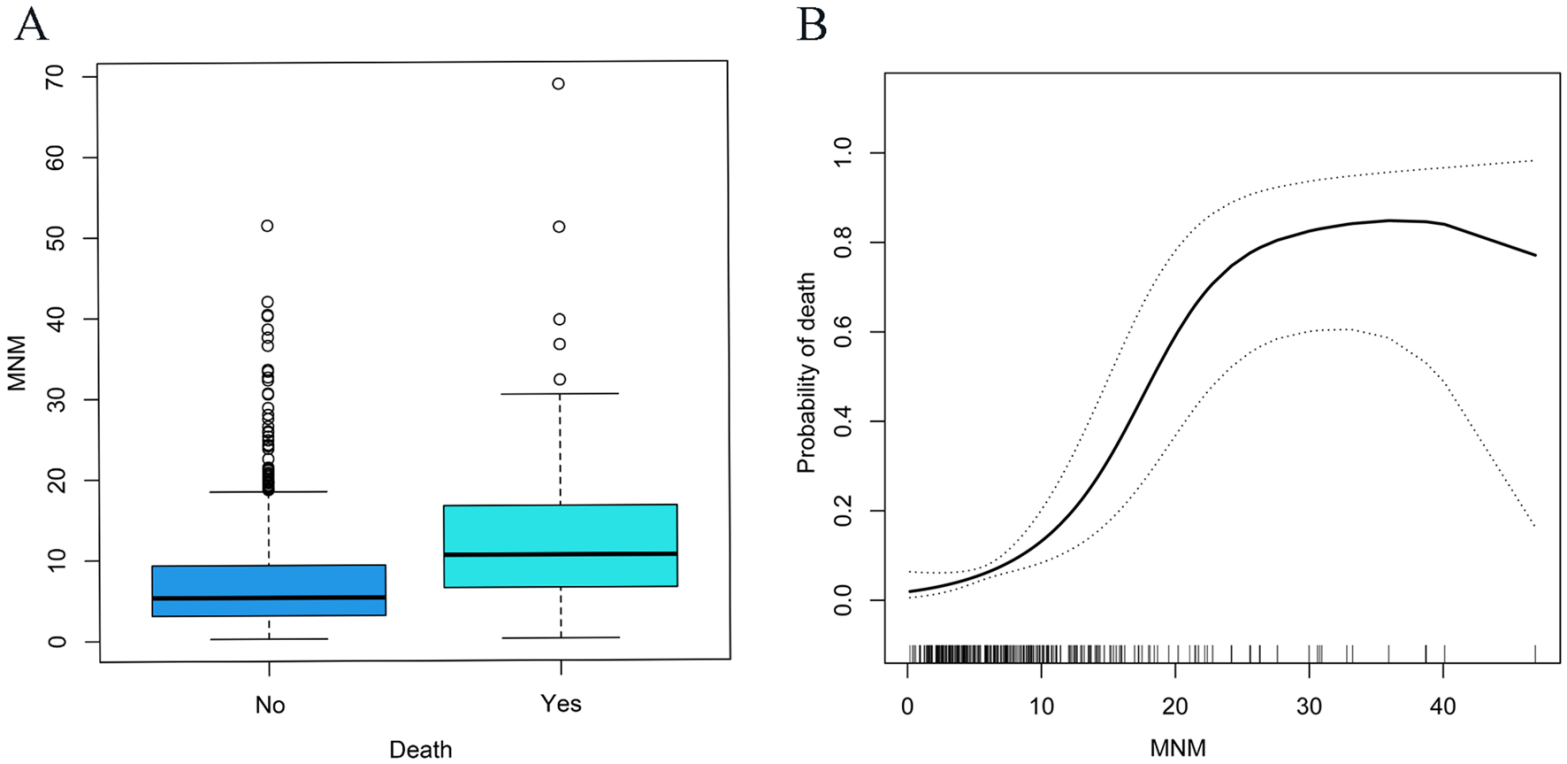
MNM level was positively associated with in-hospital death in patients with aSAH. **A** As seen from the box plots, the MNM distribution differed for the death and survival groups. **B** There was a nonlinear relationship between the probability of in-hospital death and the level of MNM

**Fig. 2:**
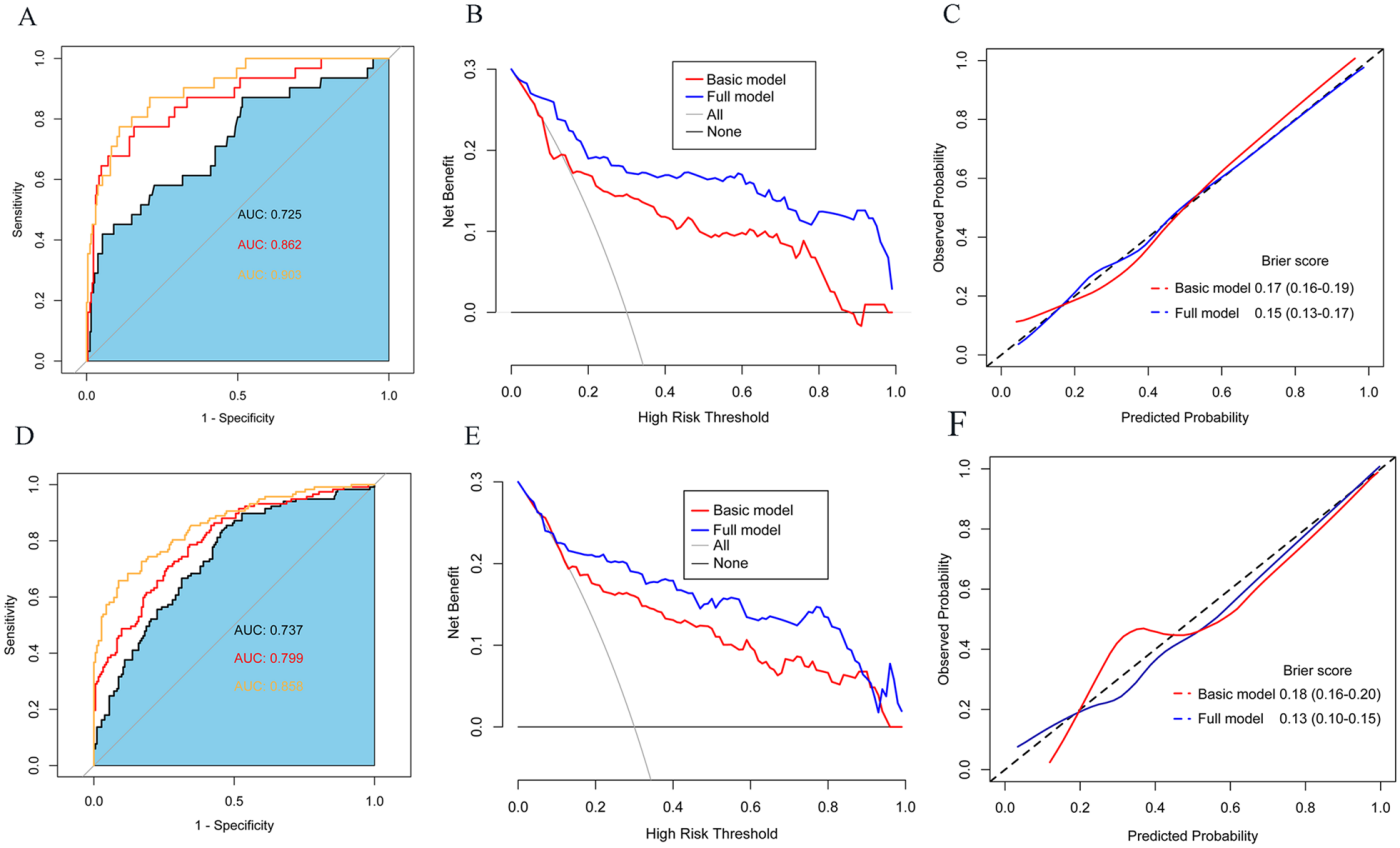
**A** ROC curve analysis of MNM and basic and full models for in-hospital death in the development cohort. **B** The DCA curve indicates that the full model shows a higher net benefit than the basic model in the development cohort. **C** Calibration curves of the basic model and full model prediction of in-hospital death in the development cohort. **D** ROC curve analysis of MNM and basic and full models for in-hospital death in the validation cohort. **E** The DCA curve indicates that the full model shows a higher net benefit than the basic model in the validation cohort. **F** Calibration curves of the basic model and full model prediction of in-hospital death in the validation cohort

The development cohort showed that MNM was closely related to in-hospital death in patients with aSAH based on univariate logistic regression (odds ratio [OR], 1.03; 95% confidence interval [CI] 1.01–1.06; Table 3). According to multivariate logistic regression analysis, higher MNM was still associated with in-hospital death (OR, 1.04; 95% CI 1.01–1.07; Table 4) after controlling for other risk factors. As MNM increased, the likelihood that a patient would die during hospitalization increased. The median MNM of the dead patients was significantly higher than that of the alive patients (Fig. 1A). Smoothed plots suggest a nonlinear positive relationship between MNM and the incidence of in-hospital death. The larger the MNM, the higher the probability of death (Fig. 1B). The AUC of MNM for in-hospital death was 0.725 (95% CI 0.640–0.846; Fig. 2A).

**Table 3:** Univariable analysis of predictive factors for death in hospital

Variable	Development cohort		Validation cohort	
	Odds ratio (95% CI)	*p* value	Odds ratio (95% CI)	*p* value
Sex	1.29 (0.77, 2.16)	0.335	0.76 (0.36, 1.60)	0.471
Age	0.99 (0.97, 1.01)	0.271	0.97 (0.96, 0.98)	< 0.001
Level on GCS score				
Mild (13–15 points)	1 [Reference]	1 [Reference]	1 [Reference]	1 [Reference]
Moderate (9–12 points)	0.48 (0.23, 1.00)	< 0.001	0.11 (0.04, 0.30)	< 0.001
Severe (3–8 points)	0.19 (0.11, 0.33)	0.017	0.22 (0.08, 0.63)	0.005
MAP, mmHg	0.99 (0.98, 1.00)	0.069	0.98 (0.98, 0.99)	< 0.001
Hypertension (Yes vs No)	0.65 (0.40, 1.07)	0.089	0.12 (0.07, 0.20)	< 0.001
Diabetes (Yes vs No)	0.57 (0.26, 1.27)	0.171	0.27 (0.09, 0.80)	0.019
Hunt–Hess grade				
I, II, III	1 [Reference]	1 [Reference]	1 [Reference]	1 [Reference]
IV, V	1.25 (0.45, 3.45)	< 0.001	1.05 (1.01, 1.09)	0.002
mFS grade				
0, I, II	1 [Reference]	1 [Reference]	1 [Reference]	1 [Reference]
III, IV	1.42 (1.70, 2.87)	< 0.001	1.25 (1.14, 1.43)	< 0.001
NIHSS score	1.15 (1.04, 1.27)	< 0.001	1.05 (1.02, 1.08)	< 0.001
Smoking	5.47 (0.74, 40.32)	0.095	0.11 (0.01, 1.84)	0.126
Drink abuse	4.03 (0.54, 29.89)	0.173	0.11 (0.01, 1.84)	0.126
Blood glucose	0.82 (0.76, 0.87)	< 0.001	1.11 (1.01, 1.22)	0.029
Serum calcium level	0.44 (0.08, 2.56)	0.361	1.60 (0.09, 30.07)	0.753
Inflammatory index				
Leukocyte count	0.88 (0.85, 0.92)	< 0.001	1.20 (1.11, 1.30)	< 0.001
Neutrophil count	0.88 (0.84, 0.92)	< 0.001	1.16 (1.07, 1.26)	< 0.001
Lymphocyte count	0.96 (0.79, 1.16)	0.653	1.28 (1.00, 1.64)	0.050
Monocyte count	0.26 (0.15, 0.47)	< 0.001	6.73 (3.16, 14.34)	< 0.001
Platelet count	1.00 (1.00, 1.00)	0.700	1.00 (1.00, 1.01)	0.583
NLR	0.96 (0.93, 0.99)	< 0.001	0.81 (0.77, 0.85)	< 0.001
MLR	0.31 (0.17, 0.57)	< 0.001	0.05 (0.03, 0.11)	< 0.001
PLR	1.00 (1.00, 1.00)	0.992	1.00 (0.99, 1.00)	0.073
NWR	0.05 (0.00, 0.78)	0.032	0.07 (0.04, 0.11)	< 0.001
MNM	1.03 (1.01, 1.06)	< 0.001	1.13 (1.08, 1.18)	< 0.001

*GCS* Glasgow Coma Scale, *MAP* mean arterial pressure, *mFS grade* modified Fisher grade, *NIHSS* National Institutes of Health Stroke Scale, *NLR* neutrophil-to-lymphocyte ratio, *MLR* monocyte-to-lymphocyte ratio; *PLR* platelet-to-lymphocyte ratio, *NWR* neutrophil-to-leukocyte ratio, *MNM* multiplication to neutrophil and monocyte counts

**Table 4:** The Multivariate regression analysis for predicting death in hospital

Variable	Development cohort		Validation cohort	
	Odds ratio (95% CI)	*p* value	Odds ratio (95% CI)	*p* value
Hunt–Hess grade		0.035		0.002
I, II, III	1 [Reference]		1 [Reference]	
IV, V	1.92 (1.05, 3.52)		5.31 (1.82, 15.54)	
mFS grade		0.008		0.037
0, I, II	1 [Reference]		1 [Reference]	
III, IV	2.09 (1.21, 3.62)		4.25 (1.25, 14.47)	
NIHSS score	1.03 (1.01, 1.05)	0.010	1.07 (1.03, 1.11)	0.001
Blood glucose	1.13 (1.05, 1.22)	0.001	1.15 (1.02, 1.30)	0.027
MNM	1.04 (1.01, 1.07)	0.004	1.13 (1.07, 1.19)	< 0.001

*mFS grade modified Fisher grade, NIHSS National Institutes of Health Stroke Scale, MNM* multiplication to neutrophil and monocyte counts

### A predictive model for in-hospital death

Two multivariate logistic regression models were introduced in the development cohort to predict in-hospital death (basic and full models, respectively) (Table 3). The basic model included Hunt–Hess grade, mFS grade, NIHSS score, and glucose, whereas the full model included these factors and MNM. The full model showed a higher area under the curve (0.903 vs. 0.862) and higher specificity (90.31% vs. 83.45%) compared with the basic model. However, the sensitivity of both models in predicting in-hospital death was similar (68.18% vs. 65.50%) (Fig. 2A). DCA curve demonstrated that the two models showed compelling clinical values. The full model showed a preferred net benefit over the basic model with a threshold probability in the range of 10% and 90% (Fig. 2B). Brier scores and calibration curves for the full model used to predict in-hospital death show better consistency than the basic model (Fig. 2C).

**Fig. 3:**
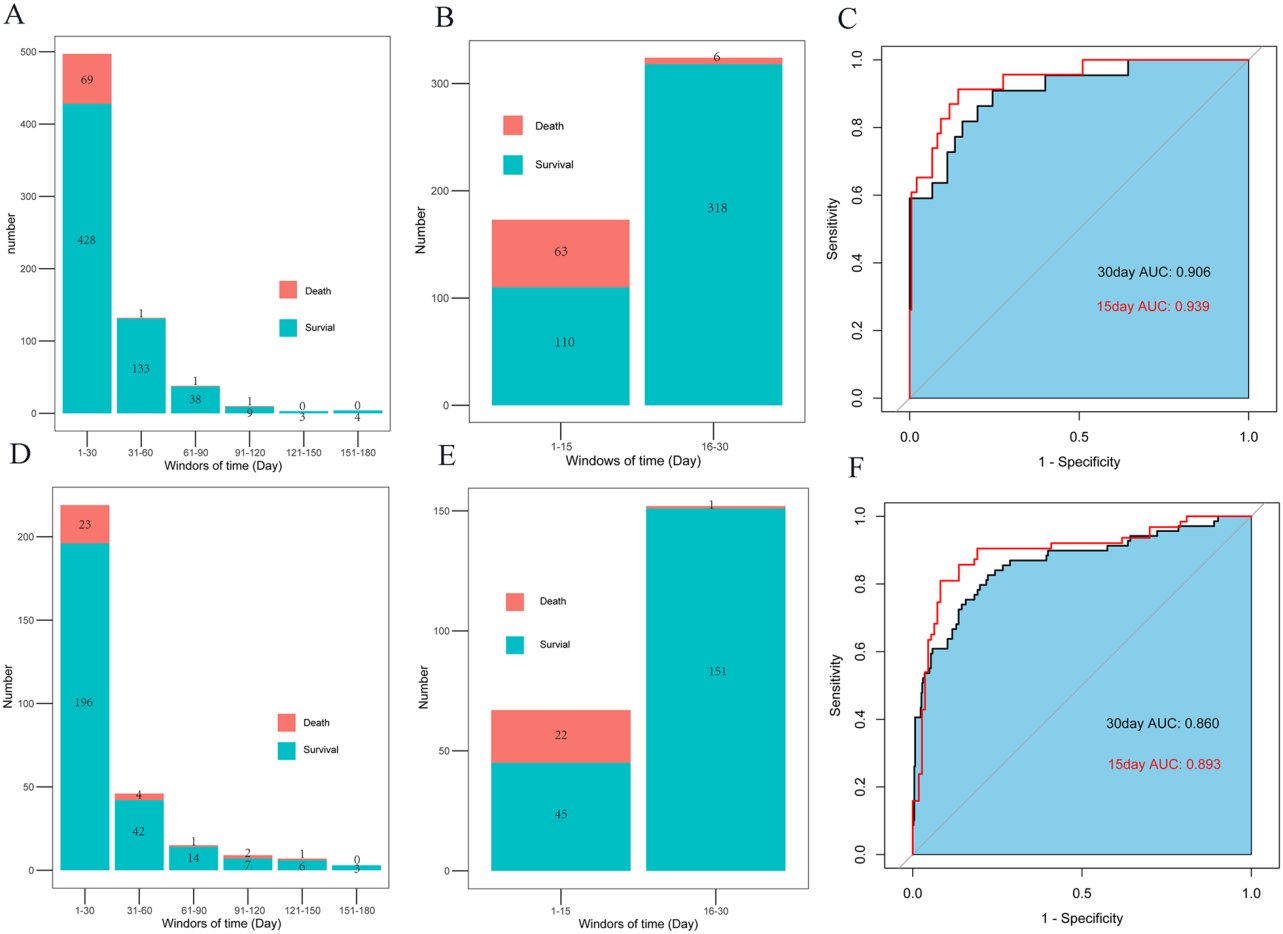
**A** Distribution of patients with different hospitalization time windows in the development cohort. **B** Distribution of patients within the hospitalization time windows of 1–15 days and 16–30 days in the development cohort. **C** AUC of the full model predicts patient in-hospital death within 30 days and 15 days in the development cohort. **D** Distribution of patients with different hospitalization time windows in the validation cohort. **E** Distribution of patients within the hospitalization time windows of 1–15 days and 16–30 days in the validation cohort. **F** AUC of the full model predicts patient in-hospital death within 30 days and 15 days in the validation cohort

Due to the large duration of hospital stay, the length of hospital stay ranged from 1 to 180 days and was divided into six periods to more accurately explore the predictive value of the full model in different time windows (Fig. 3A). Sixty-nine patients (95.83%) died within 30 days, while only one person died within 31–60 days, 61–90 days, and 91–120 days respectively. None of the patients died after 120 days. The full model also demonstrated an excellent ability to predict in-hospital death within the 30 day range (AUC, 0.906; specificity, 81.16%; sensitivity, 72.43%; Fig. 3B). Patients’ conditions at 30 days were further divided into two intervals (Fig. 3C). The full model showed better predictive power in the 1–15 days range (AUC, 0.939; specificity, 84.32%; sensitivity, 82.18%; Fig. 3C).

### External validation

In the validation cohort, MNM was strongly associated with in-hospital death in patients with aSAH based on univariate logistic regression (OR, 1.13; 95% CI 1.08–1.18; Table 3). After adjustment for other independent risk factors, higher MNM was still associated with in-hospital death (OR, 1.13; 95% CI 1.07–1.19; Table 4).

The prediction ability of the full model for in-hospital death was better than that of the basic model (AUC, 0.858 [95% CI 0.826–0.891] vs. 0.799 [95% CI 0.768–0.838]; Fig. 2D). DCA curve showed that both models had significant clinical application value and the full model showed better net benefit than the basic model (Fig. 2E). Brier scores and calibration curves of the full model agree better than the basic model between predicted and observed values (Fig. 2F). A subdivision of the hospitalization time window showed that the predictive ability of the full model over 15 days was superior to that over 30 days (AUC, 0.893 vs. 0.860; Fig. 3F).

**Fig. 4:**
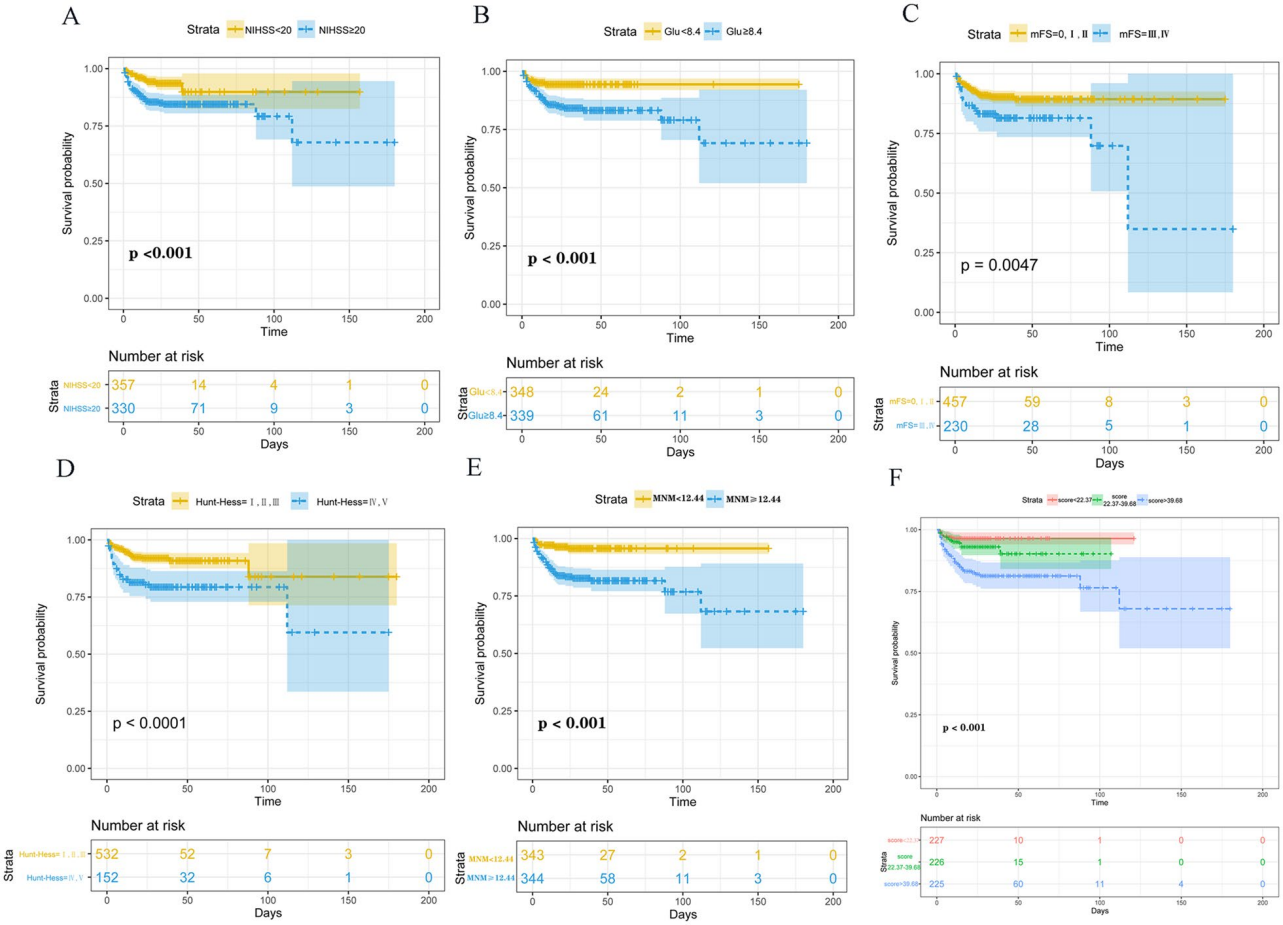
The Kaplan–Meier plot of different variables (**A**) Higher NIHSS score association with a higher in-hospital death in patients with aSAH. **B** Elevated glucose is associated with a higher in-hospital death in patients with aSAH. **C** Higher mFS grade association with a higher in-hospital death in patients with aSAH. **D** A higher Hunt–Hess grade is associated with a higher in-hospital death in patients with aSAH. **E** Higher MNM is associated with higher in-hospital death in patients with aSAH. **F** A higher total score is associated with higher in-hospital death in patients with aSAH

ROC analysis determined that the optimal cutoff value threshold of MNM for in-hospital death was 12.44. The Kaplan–Meier plot demonstrated a higher in-hospital death in patients with aSAH when admission MNM ≥ 12.44 than in patients with MNM < 12.44 (Fig. 4E). Similarly, higher Hunt–Hess grade, mFS grade, NIHSS score, and elevated glucose were associated with a higher in-hospital death in patients with aSAH (Fig. 4A–D).

**Fig. 5:**
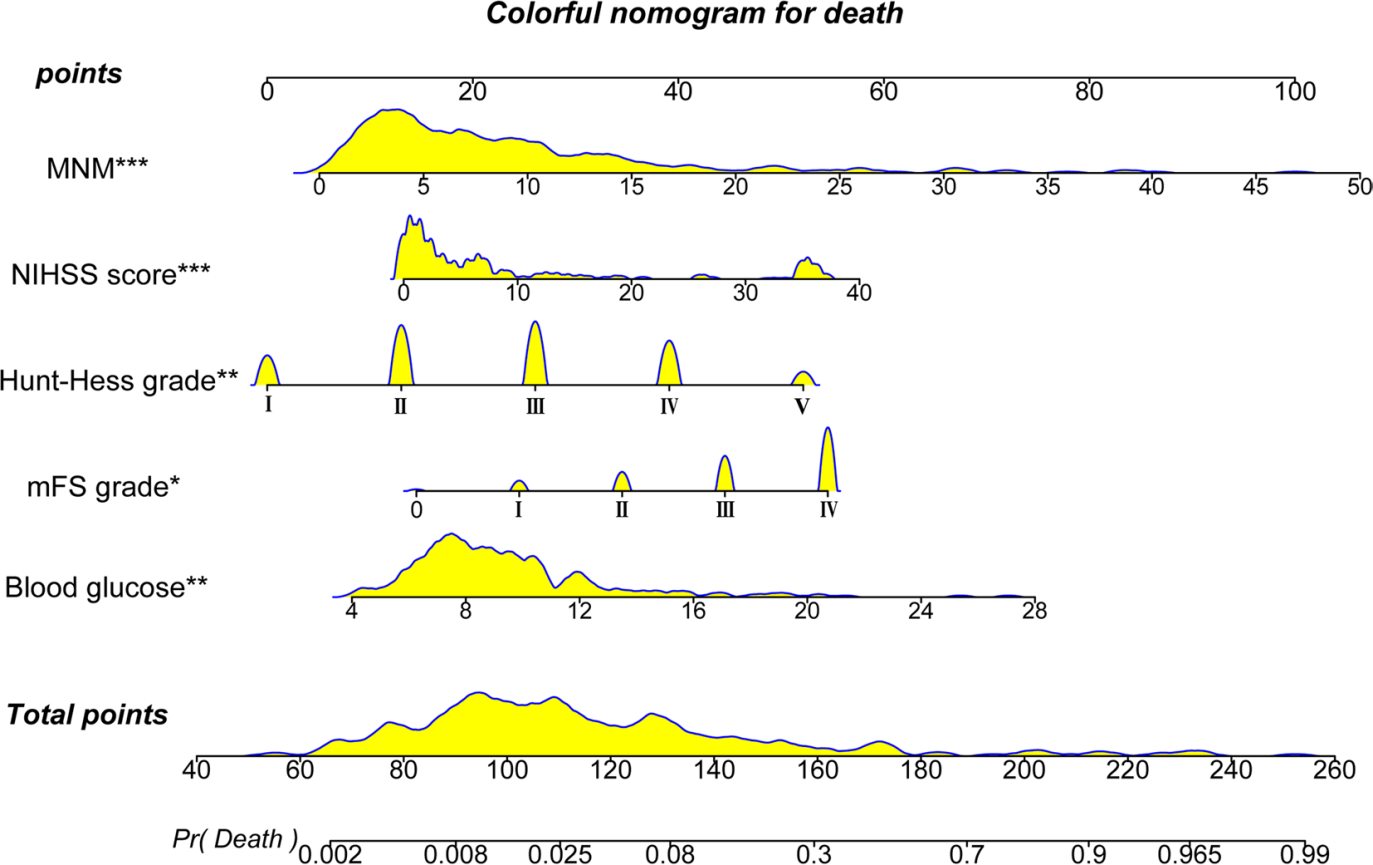
The nomogram constructed from the full model was used to predict in-hospital death. Nomogram is performed according to the great influence of each influence factor in the model on the outcome variable (the magnitude of the regression coefficient), and the value of each influence factor is assigned a score for each layer, then the score is calculated as the total score, and finally, the predicted probability of individual events is calculated by the transformation relationship between the total score and the probability function of the outcome event

**Fig. 6:**
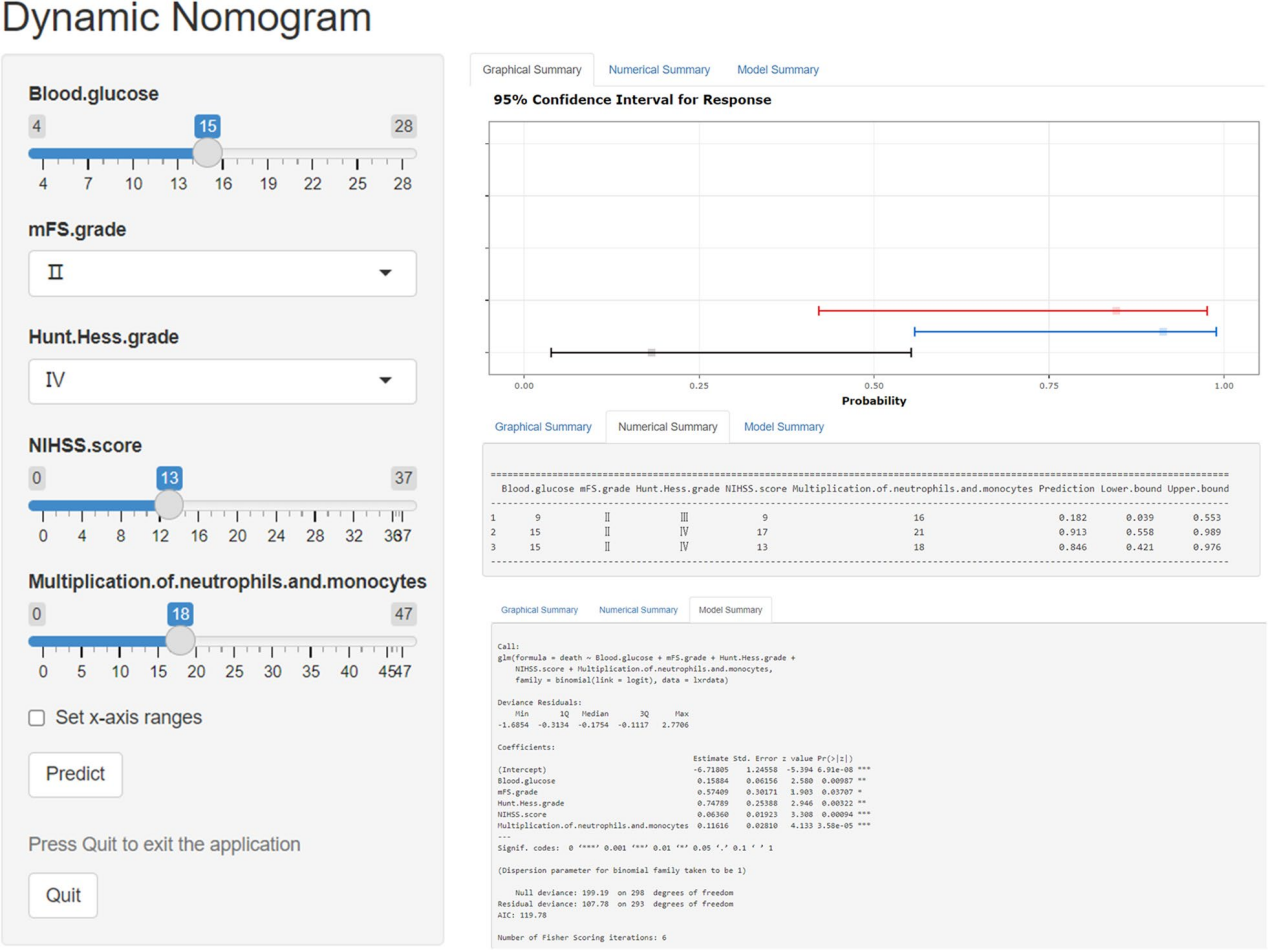
A dynamic nomogram is deployed on the shinyapps.io platform (https://dzzhuang.shinyapps.io/outcome_of_asah/). Users can calculate the probability of a poor prognosis for aSAH patients by submitting five related traits online via mobile phone or computer

To facilitate clinical application, we created a nomogram based on several clinical indicators for predicting the likelihood of death during hospitalization in patients with aSAH (Fig. 5). The nomogram translates the risk of each indicator into numerical values. The probability corresponding to the sum of the values of each indicator is the likelihood of patient death. When the total score was ≥ 39.68, the AUC for the nomogram’s prediction of the likelihood of death was 0.912 (95% CI 0.861–0.962), which was significantly higher for in-hospital deaths than for aSAH patients with a total score of < 39.68 (Fig. 4F). Finally, we uploaded the nomogram generation online tool to the shinyapps.io platform (https://dzzhuang.shinyapps.io/outcome_of_asah/). Clinicians can submit the 5 metrics in the full model to the appropriate text box for calculation via the web (Fig. 6). The results will show the likelihood of a patient dying during hospitalization, 95% confidence intervals, and other parameters.

## Discussion

Based on previous studies, our study is the first to suggest the priori value of elevated MNM at admission for in-hospital death in patients with aSAH, suggesting that MNM may serve as an autonomous indicator of in-hospital death. In the developmental cohort, MNM was positively associated with the probability of in-hospital death. The likelihood of in-hospital death increased with increasing MNM. MNM was an important component of the multifactorial in-hospital death prediction model and significantly improved the predictive power of the multifactorial in-hospital death prediction model. MNM also showed good predictive performance in the validation cohort.

In contrast, NLR is a popular inflammatory marker associated with poor prognosis [1, 4, 12], delayed cerebral ischemia [32], and rebleeding [13]. However, previous literature suggests that NLR is not necessarily significantly associated with functional prognosis, and its predictive value is limited [10]. In the present study, MNM had a stronger predictive correlation with in-hospital death in patients with aSAH than NLR.

The addition of MNM significantly improved the predictive power of the full model for in-hospital death. However, the full model did not apply to all patients. Our study’s hospitalization length of hospitalization in our study ranged from 1 to 180 days. Most of the patients who died were within 15 days, consistent with the clinical phenomenon. Therefore, the predictive value of the full model was better in the 15 days period than in other time windows. Although the AUC of the full model in our study was higher in the interval of 16–30 days than in the interval of 1–15 days, the predictive value of the full model is statistically controversial because the number of patients who died in the interval of 16–30 days was only 6. In conclusion, our study suggests that the full model is more applicable to patients with a hospitalization time window of 15 days.

MNM unites neutrophils and monocytes and is an important indicator of inflammation associated with prognosis, suggesting neuroinflammation as a key factor that exacerbates brain injury and leads to behavioral disturbances. On the one hand, following the occurrence of subarachnoid hemorrhage, catabolism and degradation of erythrocytes lead to progressive deposition of hemoglobin in the subarachnoid space. Increasing amounts of methemoglobin and hemoglobin activate toll-like receptor 4, which signals the initiation of an inflammatory cascade response [18, 33, 34]. On the other hand, blood components in the subarachnoid space activate microglia in the central nervous system. Activated immunomodulatory cells promote the upregulation of endothelial cell adhesion molecules and facilitate the infiltration of large numbers of neutrophils, monocytes and macrophages into the subarachnoid space [33, 35, 36]. Among them, neutrophils are the most common leukocyte type in the peripheral circulation and the fastest infiltrating inflammatory cells in the CNS after peripheral immunization. In a mouse model, it takes only 10 min after the onset of aSAH for neutrophils to invade the CNS, distribute in the microvasculature, and even infiltrate the brain parenchyma [37, 38]. The binding of neutrophils to vascular endothelial cells is essential for its accumulation at the injury site. This effect leads to the opening of inter-endothelial cell junctions and increased permeability, which promotes neutrophil migration to the injury site [39, 40]. Pathologically, neutrophil adhesion to the vascular endothelium can lead to acute endothelial injury. In severe cases, increased intravascular neutrophil concentrations can restrict blood flow, block blood vessels, cause ischemia, and ultimately aggravate brain injury [41].

In contrast to neutrophils, activation of monocytes is an early response of the immune system to brain tissue injury. In the acute phase of brain injury, monocytes are involved in tissue repair and as antigen-presenting cells in the communication of the innate and acquired immune system [10]. In clinical studies and mouse models, the brain is infiltrated with neutrophils and monocytes after the onset of aSAH. These studies suggest that monocytes proliferate rapidly after aneurysm rupture in the early stages of aSAH, infiltrating brain tissue and transforming into a proinflammatory phenotype [10, 42]. The mechanisms by which neutrophils and monocytes induce neuroinflammation are complex. It has been shown that neutrophils and monocytes can also be activated by microglia. aSAH similarly induces activation of microglia recruiting monocytes to differentiate into macrophages by secreting large amounts of proinflammatory factors (e.g., interleukin-6, interleukin-1β, and tumor necrosis factor-α) [43]. Similarly, early activation of astrocytes promotes neutrophil and monocyte movement and activation of macrophages, and activated macrophages activate local cellular inflammation [44, 45].

This paper developed and validated a nomogram to predict in-hospital death in patients with aSAH. The Hunt–Hess grade, mFS grade, NIHSS score, blood glucose, and MNM in this study had good predictive value in univariate and multifactorial regression analyses. The Hunt–Hess grade is a very important index to evaluate the severity of aneurysm patients and is closely related to the prognosis of patients. Similarly, Hunt–Hess grade was an important predictor of in-hospital death in aSAH patients in our study. The mFS grade reflects the severity of aSAH and is both an independent risk factor for CVS [46] and associated with the prognosis of patients with aSAH [1, 47]. The present study, in agreement with previous studies, also confirmed that the mFS grade was also an independent predictor of in-hospital death in patients with aSAH (OR, 2.09; 95% CI 1.21–3.62). the NIHSS score objectively assesses the severity of the disease based on the patient’s clinical symptoms at the time of admission to the hospital. In the current guidelines, the NIHSS score is a valid tool for assessing stroke severity [48]. In addition, elevated blood glucose is an important serologic predictor of poor prognosis and death in patients with aSAH in current and previous studies [49–51]. Notably, the hyperglycemic state after the onset of aSAH may be related to the metabolic changes induced after aSAH. More importantly, a growing body of literature suggests an important role of the inflammatory response in the prognostic development of patients with aSAH. Local and systemic inflammatory responses have an important impact on the prognosis of patients [52]. Therefore, this paper incorporates MNM as a novel inflammatory index with independent predictive value into the nomogram, which makes the predictive model more powerful and convincing.

## Limitations

Despite these results of the study, some limitations need to be considered. First, we used data from two units and external validation to minimize the effect of selection bias, but a prospective multicenter cohort study is more convincing. In addition, this study did not include other inflammatory markers such as c-reactive protein and interleukin-6. Finally, the World Federation of Neurosurgical Societies, an important indicator of aSAH, needed to be added to our data.

## Conclusion

This study suggests that elevated MNM is associated with in-hospital death and that MNM may be a new serologic predictor of in-hospital death in patients with aSAH. This novel nomogram is a convenient tool for predicting in-hospital death in patients with aSAH.

## Data Availability

All data generated or analyzed during this study are included in this published article (and its supplementary information files).

## Abbreviations

AUC: Area under the curve
aSAH: Aneurysmal subarachnoid hemorrhage
CI: Confidence interval
CTA: Computed tomography angiography
CVS: Cerebral vasospasm
DCA: Decision curve analysis
DSA: Digital subtraction angiography
GCS: Glasgow Coma Scale
MAP: Mean arterial pressure
mFS grade: Modified fisher grade
MLR: Monocyte-to-lymphocyte ratio
MNM: Multiplication to neutrophil and monocyte counts
mRS: Modified Rankin Scale
NIHSS: National Institutes of Health Stroke Scale
NLR: Neutrophil-to-lymphocyte ratio
NWR: Neutrophil-to-leukocyte ratio
OR: Odds ratio
PLR: Platelet-to-lymphocyte ratio
ROC: Receiver operating characteristic
